# Inhibition of autophagy enhances the sensitivity of ciclopirox olamine in human chronic myelogenous leukemia K562 cells

**DOI:** 10.7150/ijms.114452

**Published:** 2025-09-12

**Authors:** Hongyu Zhou, Hongyan Chen, Kaitao Luo, Lingmei Kong, Yan Li

**Affiliations:** 1 School of Pharmaceutical Science & Yunnan Key Laboratory of Pharmacology for Natural Products, Kunming Medical University, Kunming 650500, Yunnan, China.; 2 Key Laboratory of Medicinal Chemistry for Natural Resource, Ministry of Education; Yunnan Key Laboratory of Research and Development for Natural Products; School of Pharmacy, Yunnan University, Kunming 650500, China.; 3 State Key Laboratory of Phytochemistry and Plant Resources in West China, Kunming Institute of Botany, Chinese Academy of Sciences, Kunming 650201, Yunnan, China.; 4 Yunnan College of Modern Biomedical Industry, Kunming Medical University, Kunming 650500, Yunnan, China.

**Keywords:** ciclopirox olamine, leukemia, autophagy, AMP-activated protein kinase (AMPK), unc-51 like autophagy activating kinase 1 (ULK1)

## Abstract

Hematological malignancies, such as leukemia and multiple myeloma, are heterogeneous blood diseases that affect blood, lymphoid and other related tissues. Ciclopirox olamine (CPX), which is an antifungal agent, was identified as a promising anti-cancer candidate drug recently, especially in hematologic malignancy. However, CPX resistance in hematologic malignancy and the underlying molecular mechanisms are largely unknown. Our present study found that different leukemia cells exhibited different apoptotic responses to CPX. CPX effectively induced mitochondrial-mediated apoptosis in adult acute myeloid leukemia OCI-AML2 cells. However, in human chronic myelogenous leukemia K562 cells, CPX induced little apoptosis. Further study showed that CPX inhibited autophagy in OCI-AML2 cells, but induced autophagy in K562 cells. Notably, compared with CPX alone, the combination of CPX with the autophagy inhibitor chloroquine (CQ) or Bafilomycin A1 (Baf-A1) significantly enhanced pro-apoptotic activity of CPX, suggesting that CPX-induced autophagy is cytoprotective in K562 cells. Mechanism study showed that CPX induced autophagy by activating AMP-activated protein kinase (AMPK)/Unc-51 like autophagy activating kinase 1 (ULK1) signaling. Ectopic expression of dominant negative AMPKα or pharmacological inhibition of AMPK with its inhibitor compound C partially prevented from CPX inducing autophagy and re-sensitized K562 cells to CPX-induced apoptosis. Taken together, our present study demonstrates that AMPK-ULK1-mediated autophagy protects human chronic myelogenous leukemia K562 cells from CPX-induced apoptosis. To improve the clinical therapeutic efficacy of CPX in chronic myelogenous leukemia, the combination therapy of CQ and CPX might be a worthwhile alternative option.

## Introduction

Leukemia is a type of malignant clonal disease of hematopoietic stem cells, with high heterogeneity and poor prognosis 1. Based on the speed of disease development and the type of blood cell involved, leukemia is classified into four main types, they are acute lymphocytic leukemia (ALL), acute myelogenous leukemia (AML), chronic lymphocytic leukemia (CLL) and chronic myelogenous leukemia (CML) 2. The treatment approach for leukemia depends on its type and how aggressive the leukemia is. Despite several advances achieved in leukemia treatment, including chemotherapy, targeted therapy and immunotherapy, the rate of leukemia treatment failure and recurrence remains high 3, 4. Drug resistance is a major obstacle in successful treatment of leukemia 2, 5. Therefore, a better understanding of mechanisms that are related to leukemia resistance will be helpful to overcome drug resistance thereby improving the outcome in the treatment of leukemia 6.

Autophagy is an evolutionarily conserved catabolic process and plays an essential role in cell homeostasis 7, 8. During autophagy, cytoplasm, abnormal proteins and damaged organelles are sequestered into autophagosomes, which are double-membrane vesicles. The autophagosomes fuse with lysosomes to form autolysosomes and then the sequestered contents inside of the autolysosomes are digested by a series of lysosomal proteases and lysosomal hydrolases. Finally, the resulting macromolecules and monomer molecules are recycled into the cytoplasm for reuse 9, 10. Evidence from various studies has shown that dysfunctional autophagy is closely related to various pathological conditions such as cancer, neurodegeneration and metabolic disorders 11. Autophagy functions like a dual-edged sword and the role of autophagy in cancer is complicated. Depending on the cellular context and the stimulus, autophagy can either promote or inhibit cancer cell survival and progression 12. Due this property, the aberrant autophagy can either accelerate or inhibit drug resistance in cancer 13. In leukemia, activation of autophagy by antileukemic agents acts as a pro-survival response involved in the drug resistance of AML, such as chemotherapy drug cytarabine 6, BET inhibitors 14 and BCL2 inhibitors 15.

Autophagy is strictly regulated by various autophagy⁃related genes (ATGs), of which the ATG1 was the first ATG discovered in yeast 16-18. Unc-51-like autophagy activating kinase 1 (ULK1), a mammalian homologous of the yeast kinase ATG1, has an essential role in autophagy initiation 17, 19. AMP-activated protein kinase (AMPK) and mammalian target of rapamycin (mTOR) are two critical proteins in autophagy regulation 19-21. AMPK is an energy sensor that plays a critical role in maintaining energy homeostasis and mTOR is a central regulator of cell-growth 21. Studies showed that ULK1 is regulated via opposing phosphorylation by AMPK and mTOR 21. As a direct substrate of AMPK, ULK1 can be phosphorylated by AMPK at six different sites (S317, S467, S555, T575, S637 and S777) 21. Notably, mTORC1 phosphorylates ULK1 on S757 and this phosphorylation disrupts ULK1 and AMPK interaction 21.

Old drugs which have previously unrecognized therapeutic efficacy could be rapidly repurposed for new application because they have been found to have prior pharmacokinetic property and safety in humans 22, 23. The antifungal agent ciclopirox olamine (CPX), which is widely used for the topical treatment of cutaneous fungal infections, was recently identified as a promising anti-cancer candidate drug, especially in hematologic malignancy 22-26. As an intracellular iron chelator, CPX decreases cell growth and viability of malignant leukemia, myeloma as well as primary AML cells 26. Mechanistically, CPX bound intracellular iron and inhibited the iron-dependent enzyme ribonucleotide reductase at concentrations associated with its cytotoxicity 26. In 2013, the first-in-human phase I clinical trial (NCT00990587) of CPX in patients with relapsed or refractory malignant hematologic diseases was conducted 24. The study showed that oral administration of CPX was well tolerated in all patients 24. However, hematologic improvement was observed only in two patients, indicating that some patients might have intrinsic resistance to CPX, which prompted us to further explore the underlying mechanism and investigate the strategy to improve the anti-cancer effect of CPX. Our present study found that different leukemia cells exhibited different apoptotic responses to CPX. CPX effectively induced mitochondrial-mediated apoptosis in adult acute myeloid leukemia OCI-AML2 cells, but did not induce apoptosis in human chronic myelogenous leukemia K562 cells. Further study showed that CPX inhibited autophagy in OCI-AML2 cells, but induced autophagy in K562 cells. Notably, compared with CPX alone, the combination of CPX with the autophagy inhibitor chloroquine (CQ) or Bafilomycin A1 (Baf-A1) significantly enhanced pro-apoptotic activity of CPX, suggesting that CPX-induced autophagy is cytoprotective in K562 cells. Mechanism study showed that CPX induces autophagy by activating AMP-activated protein kinase (AMPK)/Unc-51 like autophagy activating kinase 1 (ULK1) signaling. Ectopic expression of dominant negative AMPKα or pharmacological inhibition of AMPK with its inhibitor compound C partially prevented from CPX inducing autophagy and re-sensitized K562 cells to CPX-induced apoptosis. Taken together, our present study demonstrates that AMPK-ULK1-mediated autophagy protects human chronic myelogenous leukemia K562 cells from CPX-induced apoptosis. To improve the clinical therapeutic efficacy of CPX in chronic myelogenous leukemia, the combination therapy of CQ and CPX might be a worthwhile alternative option.

## Materials and Methods

### Cell lines and cell culture

Human chronic myelogenous leukemia K562 cell, human promyelocytic leukemia HL-60 cell, human acute T cell leukemia Jurkat cell and human B acute lymphoblastic leukemia cell line RS4-11 were obtained from the Shanghai Institute of Biochemistry and Cell Biology, Chinese Academy of Sciences (Shanghai, China). Cells authenticated by suppliers were expanded and stored in liquid nitrogen once receiving each cell line. Adult acute myeloid leukemia OCI-M2 and OCI-AML2 leukemia cell lines were kindly gifted from Dr. Mark D. Minden (Princess Margaret Cancer Centre, University Health Network, Toronto, Ontario, Canada). K562, HL-60, Jurkat and RS4-11 cells were cultured in RPMI 1640 (Sigma, St. Louis, MO, USA) supplemented with 10% fetal bovine serum (HyClone, Logan, UT). OCI-M2 and OCI-AML2 cells were cultured in Alpha-MEM (Gibco, USA) supplemented with 10% FBS. All cell lines were cultured according to the suppliers' instructions and passaged no more than two months.

### Chemicals and reagents

CPX was purchased from Sigma (Purity >98%; St. Louis, MO, USA). The AMPK inhibitor compound C, the autophagy inhibitors, Chloroquine (CQ) and Bafilomycin A1 (Baf-A1), were purchased from Selleckchem (Purity >98%; Houston, TX, USA). CellTiter 96® AQueous One Solution Cell Proliferation Assay kit was purchased from Promega (Madison, WI). Annexin V-FITC Apoptosis Detection Kit I was from BD Biosciences (San Jose, CA). Enhanced chemiluminescence solution was from Perkin-Elmer Life Science (Boston, MA, USA). Antibodies of LC3 (Cell Signaling Technology, MA, USA) and p62 (Santa Cruz Biotechnologies, CA, USA) were used to detect the expression of autophagy biomarkers, LC3 and p62. Antibodies of phospho-S6K1 (T389), S6K1, phospho-S6 (S235/236), 4E-BP1, phospho-4E-BP1 (S65), phospho-ULK1 (S555), phospho-ULK1 (S757), phospho-ACC (S79) and ACC, which were purchased from Cell Signaling Technology (Boston, MA, USA), were used to determine the phosphorylation and the total protein expression level of the key proteins in AMPK-ULK1 and mTORC1-ULK1 signaling pathways. Antibodies of PARP, caspase-9 and cleaved caspase-3 (Cell Signaling Technology, MA, USA) were used to detect the cleavage of PARP, caspase-9 and caspase-3, which are critical initiator and executioner of apoptosis. β-actin antibody, the secondary antibodies of anti-Mouse IgG-Peroxidase (A9044) and anti-Rabbit IgG-Peroxidase (A0545) were from Sigma-Aldrich (Saint-Louis, MO, USA).

### Cell viability assay

To determine the cytotoxicity of CPX, cell viability was determined by MTS assay, according to the protocol of CellTiter 96®AQueous One Solution Cell Proliferation Assay kit (Promega). Briefly, 100 μl of cell suspensions were seeded into 96-well plates (1×10^4^ cells/well) and incubated overnight. The cells were treated with different concentrations of CPX (0, 0.064, 0.32, 1.6, 8, 40 μM) for 48 h, followed by MTS assay. Briefly, 20 μl of One Solution Reagent was added into each well. After further incubation at 37°C for 1-2 h, cell viability was determined by measuring the optical density (OD) at 490 nm using a Wallac 1420 Multilabel Counter (PerkinElmer Life Sciences, Wellesley, MA). The half inhibitory concentration (IC_50_) of CPX on cell viability was determined by the relative survival curve.

### Cell morphological analysis

K562 and OCI-AML2 Cells were seeded in six well plates at a density of 3×10^5^ cells per well. The next day, the cells were treated with CPX (0, 5 and 20 μM) for 48 h. Images of the cell morphology were taken with an Olympus inverted phase-contrast microscope (Olympus, USA).

### Apoptosis assay

K562 and OCI-AML2 cells were seeded into 6-well plates (6×10^5^ cells/well) and incubated overnight. The cells were exposed to CPX or indicated compounds for 48 h. The cells were then collected and washed with cold PBS. Apoptosis assay was performed using Annexin V-FITC Apoptosis Detection Kit (BD Biosciences). In brief, cells were suspended in 100 μl of Annexin-V binding buffer, followed by incubation with FITC conjugated Annexin V and propidium iodide (PI) for 15 min at room temperature in the dark. The percentage of apoptotic cells was evaluated using a FACS Calibur flow cytometer (Becton Dickinson, San Jose, CA). Cell cycle distribution was analyzed with FlowJo 7.6.1 software (FlowJo LLC, USA). The target cell population on the FSC/SSC chart was selected and circled, then the horizontal and vertical axes was set as Annexin V-FITC and PI channel, respectively. Cells that are considered viable are FITC Annexin V and PI negative. Cells that are in early apoptosis are FITC Annexin V positive and PI negative, and cells that are in late apoptosis are both FITC Annexin V and PI positive.

### Mitochondrial membrane potential (ΔΨm) analysis

2 ml of cell suspensions were seeded into 6-well plates (2×10^5^ cells/well) and incubated overnight. The cells were then treated with indicated concentrations of CPX for 24 h. The ΔΨm was analyzed by flow cytometry using JC-1 staining. Briefly, the cells were collected, washed with PBS and incubated with 10 mM JC-1 for 30 min at 37°C in the dark. The cells were then detected using a FACS Calibur flow cytometer (Becton Dickinson, San Jose, CA). In healthy cells with normal ΔΨm, JC-1 forms aggregates with red fluorescence. As the membrane potential decreases in apoptotic cells, JC-1 becomes monomeric and only shows green fluorescence. Changes in the red-green fluorescence were used to indicate changes in ΔΨm.

### Western blot analysis

K562 and OCI-AML2 cells were seeded into 6-well plates (6×10^5^ cells/well) and incubated overnight. The cells were treated with CPX (0-20 μM) for 24 h, or with 20 μM CPX for different time periods. For experiments with autophagy inhibitors, cells were pre-incubated with or without CQ or Baf-A1 for 1 h, respectively. The cells were then treated with CPX (0, 10 and 20 μM) for 24 h. Cell lysis and immunoblotting were performed as described previously 27. In brief, the cells were washed with cold PBS and lysed in RIPA buffer. Cell lysates were sonicated for twice and then centrifuged at 13,000 rpm for 10 min. Protein concentration was determined by BCA Protein Assay Kit (Beyotime, China). Equivalent amounts of protein were separated on 8-12% SDS-polyacrylamide gel and transferred to polyvinylidene difluoride membranes (Millipore, Bedford, MA). Membranes were incubated in blocking buffer (PBS containing 0.1% Tween-20 and 5% nonfat dry milk) for 1 h at room temperature and then incubated with the primary antibodies overnight. After washing with TBST for three times, the membranes were incubated with the secondary antibodies conjugated to HRP. After washing, immunoreactive bands were visualized by using enhanced chemiluminescence solution and signal was acquired using ImageQuant LASmini 4000 (GE Healthcare).

### Acridine Orange (AO) staining assay

K562 cells were seeded into 6-well plates (6×10^5^ cells/well) and incubated overnight. The cells were treated with CPX (0, 5, 20 μM) for 24 h and stained with 1 mg/ml of AO for 15 min at 37 °C. Then the cells were collected and washed in cold PBS once. Green (510-530 nm) and red (650 nm) fluorescence emission from 10000 cells was measured with a FACS Calibur flow cytometer (Becton Dickinson, San Jose, CA). Data analysis was conducted using FlowJo 7.6.1 software.

### Transmission electron microscopy (TEM) analysis

K562 cells were treated with CPX (0, 20 μM) for 24 h, collected and fixed in 2.5% glutaraldehyde and sodium cacodylate solution for 2 h, and then fixed with 1% OsO_4_ in cacodylate buffer. These cells were then dehydrated with graded alcohol and embedded in Epon-Araldite resin. Ultrathin sections were prepared on a Leica EM UC7 ultramicrotome, contrasted with uranyl acetate and photographed with a transmission electron microscope (JME-1400HC; JEOL, Japan).

### Infection of cells with recombinant adenovirus Ad-AMPK-DN

Recombinant adenoviruses expressing dominant negative AMPKα (Ad-AMPK-DN) 28 was generously provided by Dr. Shile Huang (Louisiana State University Health Science Center, USA). The control virus encoding the green fluorescence protein (GFP) alone (Ad-GFP) was described previously 29. For experiments, K562 cells were seeded in 6-well plates at a density of 6 × 10^5^ cells/well. The next day, the cells were infected with the Ad-AMPK-DN or Ad-GFP (as a control) for 24 h at 1 of multiplicity of infection (MOI = 1). The cells were then treated with CPX (0, 10 μM) for 24 h followed by western blot analysis.

### Statistical analysis

Results are expressed as mean values ± standard deviation (mean ± SD) of at least three independent experiments. The data were analyzed by using an unpaired *t* test. A level of *P* < 0.05 was considered to be statistically significant.

## Results

### The inhibitory effect of CPX on cell viability in different human leukemia cell lines

**Figure 1:**
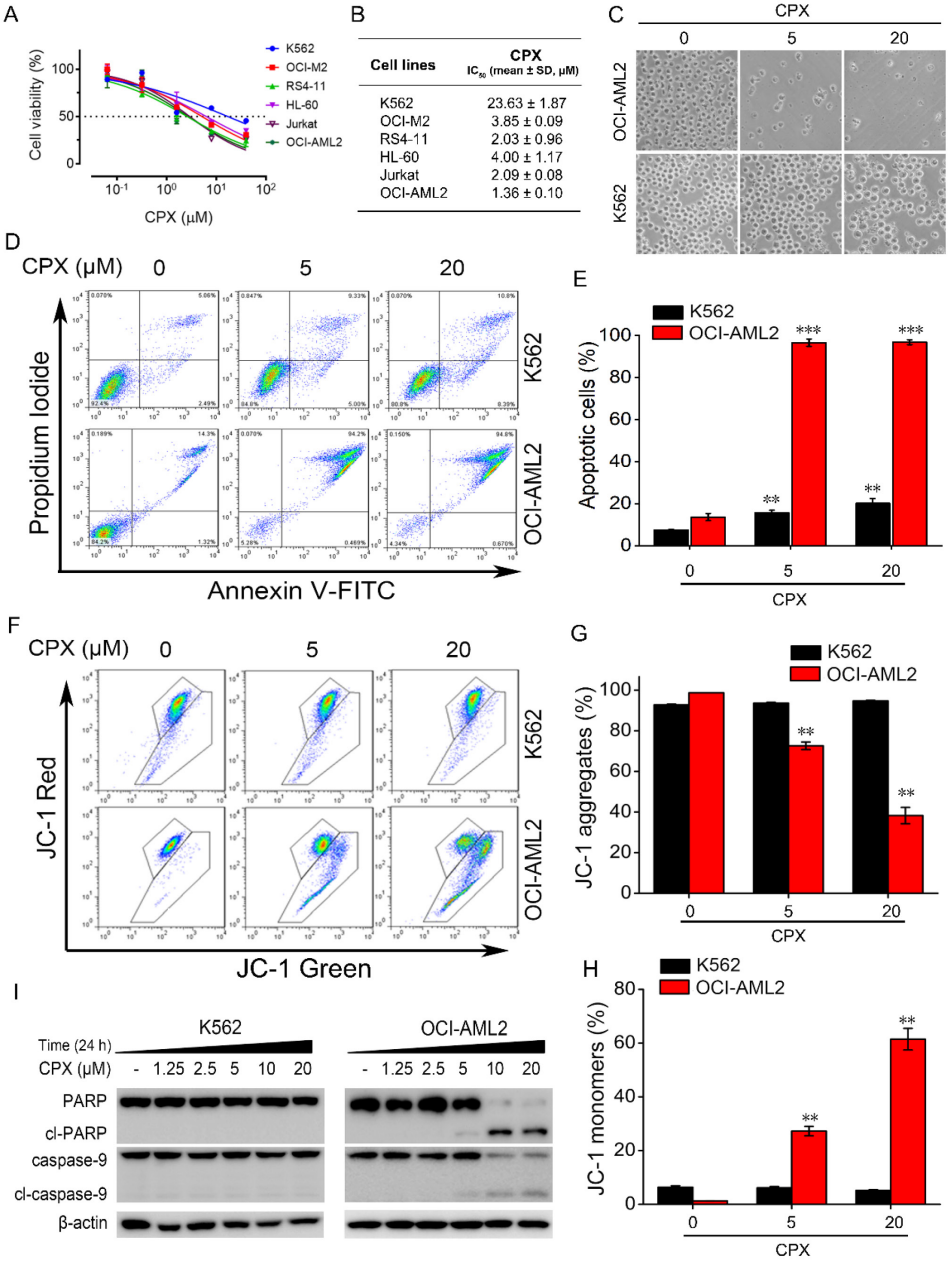
The effect of CPX on cell viability and cell apoptosis in different human leukemia cells. (A) The indicated cells were treated with CPX (0, 0.064, 0.32, 1.6, 8, 40 μM) for 48 h, cell viability was determined by MTS assay. (B) The IC_50_ values of CPX in different leukemia cell lines was calculated according to the results from MTS assay. (C and D) K562 and OCI-AML2 cells were treated with CPX (0, 5 and 20 μM) for 48 h, followed by morphological analysis (C) and apoptosis assay (D), as described in Materials and methods. (E) The percent of apoptotic cells was analyzed with FlowJo 7.6.1 software. (F) The indicated cells were treated with CPX (0, 5 and 20 μM) for 24 h. The ΔΨm was determined by flow cytometry using JC-1 staining. (G and H) The percentage of red fluorescence of JC-1 aggregates and green fluorescence of JC-1 monomers were analyzed with FlowJo 7.6.1 software. **p < 0.01, ***p < 0.001 difference versus vehicle-treated control group. (I) The indicated cells were treated with CPX (0, 5 and 20 μM) for 24 h. The cells were harvested and subjected to western blot analysis. β-actin was used as a loading control.

We initially examined the cytotoxicity of CPX in a panel of human leukemia cell lines, including human chronic myelogenous leukemia K562 cells, human promyelocytic leukemia HL-60 cells, human acute T cell leukemia Jurkat cells, human B acute lymphoblastic leukemia RS4-11 cells, adult acute myeloid leukemia OCI-M2 and OCI-AML2 cells. Cells were treated with increasing concentrations of CPX for 48 h, and cell viability was assessed by MTS assay. As shown in Figure. 1A, CPX effectively decreased the viability of these cells in a concentration-dependent manner. Among these cell lines, OCI-AML2 cells were most sensitive to CPX with an IC_50_ value of 1.36 ± 0.10 μM (Figure. 1B). By contrast, cell viability reduction by CPX was minimal in K562 cells with an IC_50_ value of 23.63 ± 1.87 μM (Figure. 1B), indicating that K562 cells were not as sensitive to CPX as the other five leukemia cell cultures (Figure. 1B). By a phase contrast microscope observation, we found that OCI-AML2 cells almost died after 5 or 20 μM concentration of CPX treatment for 48 hours (Figure. 1C). However, the morphology and the number of K562 cells were almost unaffected by the same concentration of CPX treatment for 48 hours (Figure. 1C).

### CPX effectively induced apoptosis in OCI-AML2 cells but not in K562 cells

Given our observations that OCI-AML2 cells were most sensitive to CPX, and K562 cells were most resistant to CPX among the tested leukemia cell lines, the comparative effects of CPX on the induction of apoptosis in OCI-AML2 and K562 cells were analyzed by Annexin V-FITC and PI staining. Flow cytometric analysis showed that 5 μM of CPX markedly increased the apoptotic fraction of OCI-AML2 cells, whereas CPX induce little apoptosis of K562 cells even at a concentration up to 20 μM (Figure. 1D and E). Decreased ΔΨm is considered a characteristic of mitochondrial dysfunction, which is an early event of apoptosis 30. Next, we examined the effect of CPX on ΔΨm in OCI-AML2 and K562 cells by using the JC-1 fluorescent probe. The results showed that CPX significantly diminished the ΔΨm, as evidenced by the reduced intensity of red fluorescence and increased intensity of green fluorescence (Figure. 1F-H). By contrast, the ΔΨm in K562 cells was not affected by CPX even at a concentration up to 20 μM (Figure. 1F-H), which was consistent with the results of apoptosis assay. The above data suggested that CPX induced the apoptosis of OCI-AML2 cells through the intrinsic apoptosis pathway (also known as the mitochondrial pathway).

Caspases are proteases responsible for initiating and executing the apoptotic program. Among them, caspase-9 plays a critical role in the initiation of the intrinsic apoptosis pathway 31. Therefore, we further examined the cleavage of caspases-9 and PARP, an early molecular marker of apoptosis 32. The results from western blot analysis showed that caspase-9 and PARP were all cleaved into their specific active forms by increasing concentrations of CPX in OCI-AML2 cells but not in K562 cells (Figure. 1I), demonstrating that CPX induced the mitochondrial-mediated apoptosis of OCI-AML2 cells but not K562 cells.

### CPX induced autophagy in K562 cells

The different response of OCI-AML2 and K562 cells to CPX and the poor apoptosis-inducing effect of CPX in K562 cells observed in our study prompted us to investigate the underlying mechanisms of this difference. Studies have shown that under different circumstances, autophagy may mediate apoptosis to kill cancer cells or protect cancer cells from apoptosis in response to chemotherapy 33. Based on this, we determined whether CPX triggered a cellular autophagy in K562 and OCI-AML2 cells.

**Figure 2:**
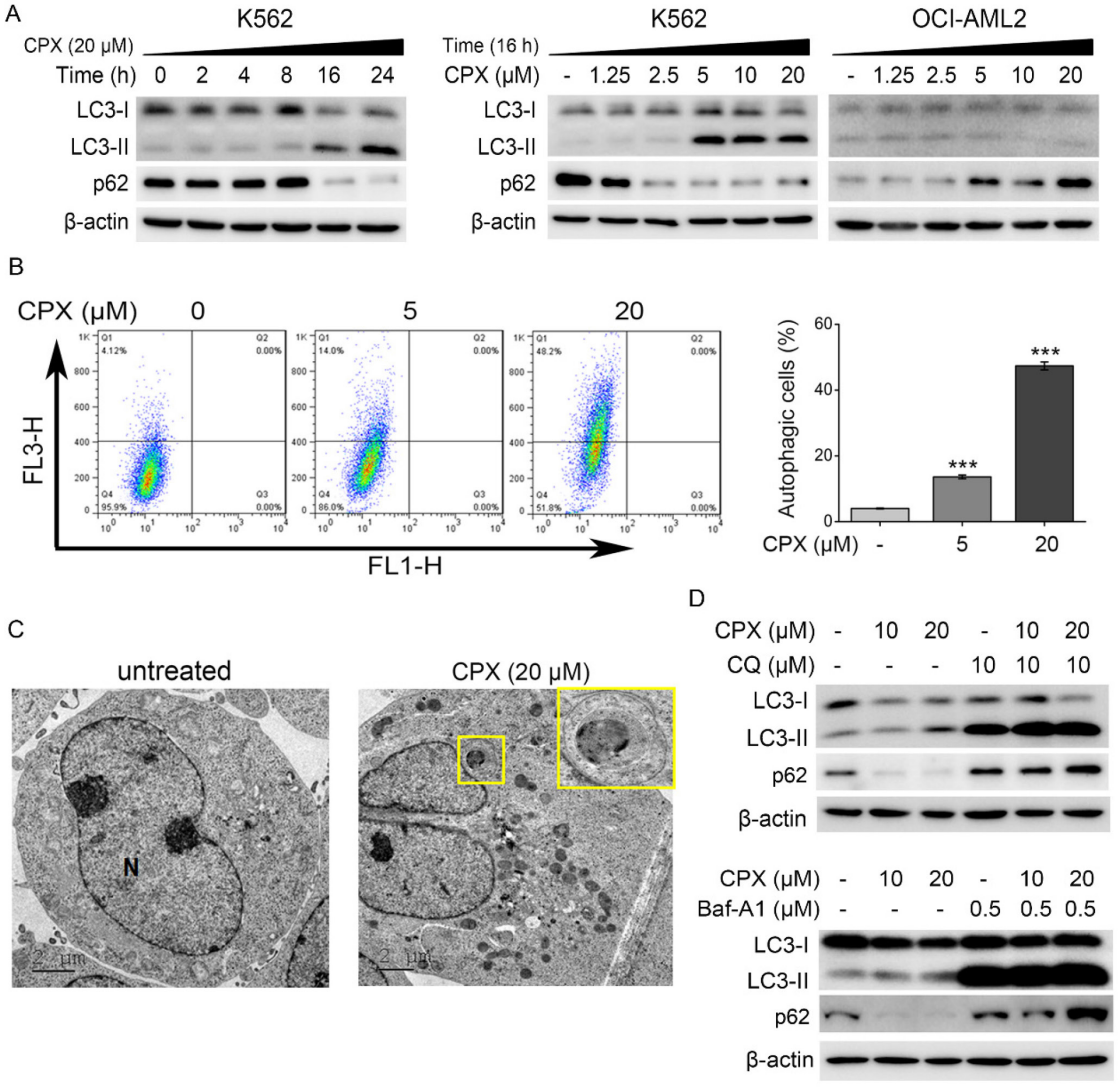
CPX induced autophagy in K562 cells. (A) Indicated cells were treated with or without 20 μM of CPX for 0, 2, 4, 8, 16 and 24 h, or treated with indicated concentrations of CPX for 24 h. The cells were harvested, lysed and subjected to western blot analysis. β-actin was used as a loading control. (B) K562 cells were treated with CPX (0, 5, 20 μM) for 24 h and stained with 1 mg/ml of OA. Green and red fluorescence emission from 10000 cells was measured with flow cytometer. Data analysis was conducted using FlowJo. Results are presented as mean ± SD (n=3). ***p < 0.001 difference versus vehicle-treated control group. (C) K562 cells were treated with CPX (0, 20 μM) for 24 h, and then collected and fixed. The TEM analysis was performed as described in Material and methods. (D) K562 cells were pretreated with indicated concentrations of CQ or Baf-A1 for 1 h, and then incubated with or without of CPX for 24 h. The cells were harvested and subjected to western blot analysis. β-actin was used as a loading control.

The conversion of LC3-I to LC3-II is the hallmark of the formation of autophagosomes during the autophagy process 34. p62/SQSTM1 is a multi-domain protein degraded by autophagy. Decreased SQSTM1 levels are associated with autophagy activation 34. Therefore, expression of LC3-II and p62 has been widely used for monitoring autophagy 34. In addition, lysosomal inhibitors, such as CQ and Baf-A1, can be used to assess increased autophagy flux based on an accumulation of LC3-II and p62 34. As shown in Figure. 2A, CPX enhanced LC3 conversion and decrease p62 protein level in time- and concentration-dependent manner in K562 cells, indicating that CPX induced autophagy in K562 cells. In contrast, LC3 conversion was decreased and p62 protein level was increased in CPX-treated OCI-AML2 cells (Figure. 2A), indicating that CPX inhibited autophagy in OCI-AML2 cells. The above data suggested that the different regulation of autophagy by CPX in K562 and OCI-AML2 cells might be related to the different sensitivity of the cells to CPX-induced cell death.

To further verify the effect of CPX on autophagy induction in K562 cells, the formation of acidic vesicular organelles (AVOs) and autophagic vesicles were further detected by AO staining and TEM analysis, respectively. The results showed that CPX effectively increased the formation of cytoplasmic AVOs in K562 cells (Figure. 2B). TEM micrographs showed that autophagic vesicles were greatly increased by CPX treatment in K562 cells (Figure. 2C). Furthermore, autophagy inhibitors, CQ and Baf-A1, were used to monitor autophagic flux under CPX treatment. The results showed that after the treatment of CPX combined with CQ or Baf-A1, the accumulation of LC3-II by CPX was further enhanced, and the reduced p62 by CPX was reversed (Figure. 2D). Taken together, the above data demonstrated that CPX induced autophagy in K562 cells.

### CPX activate ULK1 by regulating AMPK and mTORC1 signaling pathways in K562 cells

**Figure 3:**
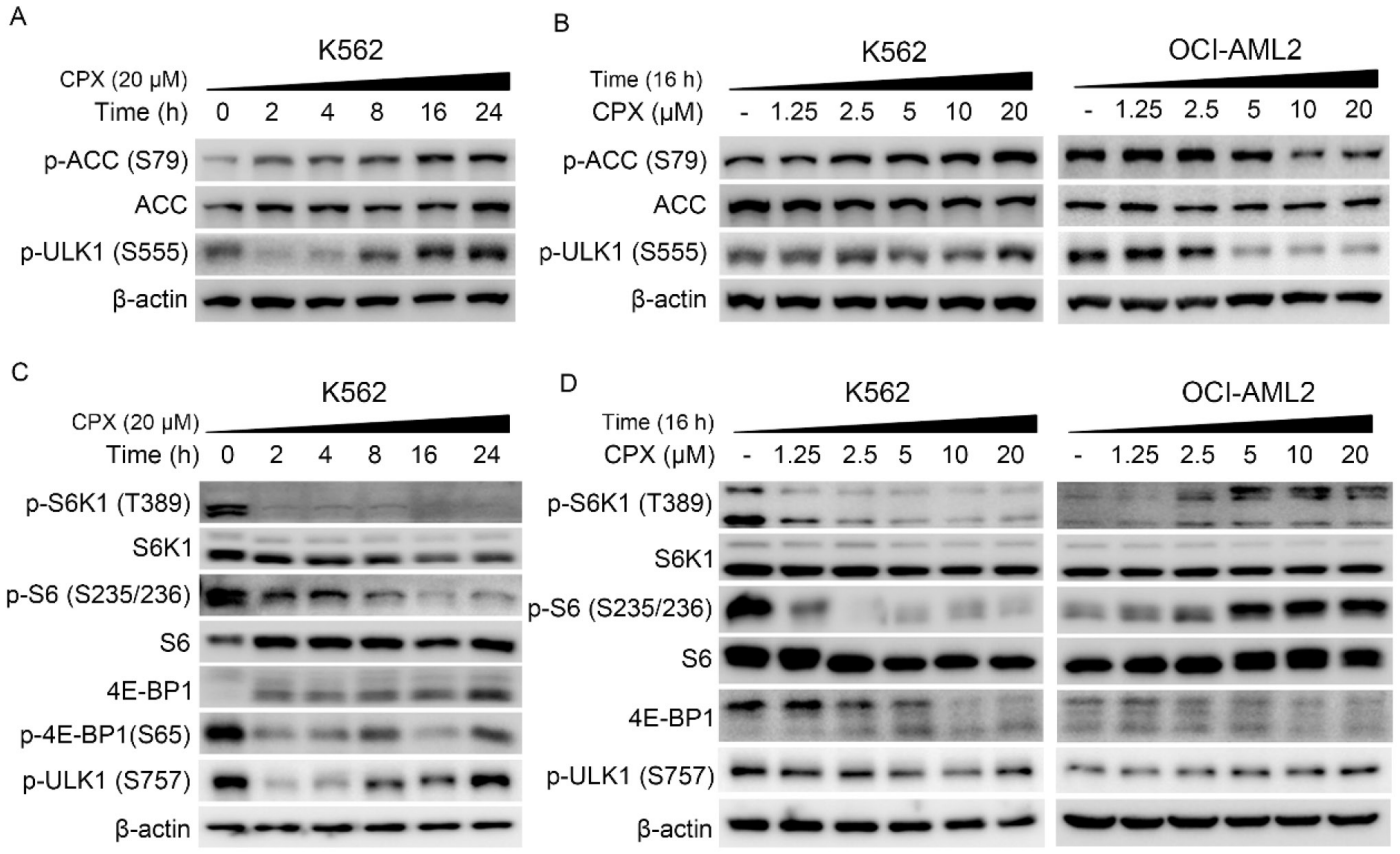
CPX induced autophagy by regulating the AMPK-ULK1 and the mTOR-ULK1 signaling pathways in K562 cells (A-D) Indicated cells were treated with or without 20 μM of CPX for 0, 2, 4, 8, 16 and 24 h (A and B), or treated with indicated concentrations of CPX for 16 h (B and D). The cells were harvested, lysed and subjected to western blot analysis. β-actin was used as a loading control.

It has been demonstrated that AMPK-ULK1 and mTOR-ULK1 pathways play a critical role in autophagy regulation. Studies showed that AMPK can activate autophagy through the activation of ULK1 by directly phosphorylating ULK1 at Ser317, Ser555 and Ser777 17, 19. In addition, mTOR activation can suppress ULK1 activity by phosphorylating ULK1 at Ser 757 and disrupting ULK1 and AMPK interaction 17. To explore the underlying mechanism by which CPX activate autophagy in K562 cells, the regulation of CPX on the AMPK/ULK1 and mTOR/ULK1 pathways was investigated. As shown in Figure. 3A and B, CPX increased the expression of phosphorylated ACC, the downstream of AMPK, and the expression of phosphorylated ULK1 (Ser 555) in K562 cells, indicating that CPX activated AMPK/ULK1 signaling in K562 cells. Meanwhile, CPX suppressed mTORC1 signaling, manifested as the decreased phosphorylation of S6K (Thr389), S6 (Ser235/236) and 4E-BP1 (Ser 65) in K562 cells (Figure. 3C and D). Consistently, the phosphorylation ULK1 at Ser 757 was decreased by CPX treatment (Figure. 3C and D). Notably, the regulation of CPX on AMPK/ULK1 and mTOR/ULK1 signaling in OCI-AML2 cells was completely opposite to that of K562 cells (Figure. 3B and D).

### Inhibition of AMPK suppressed CPX-induced autophagy and sensitized K562 cells to CPX

**Figure 4:**
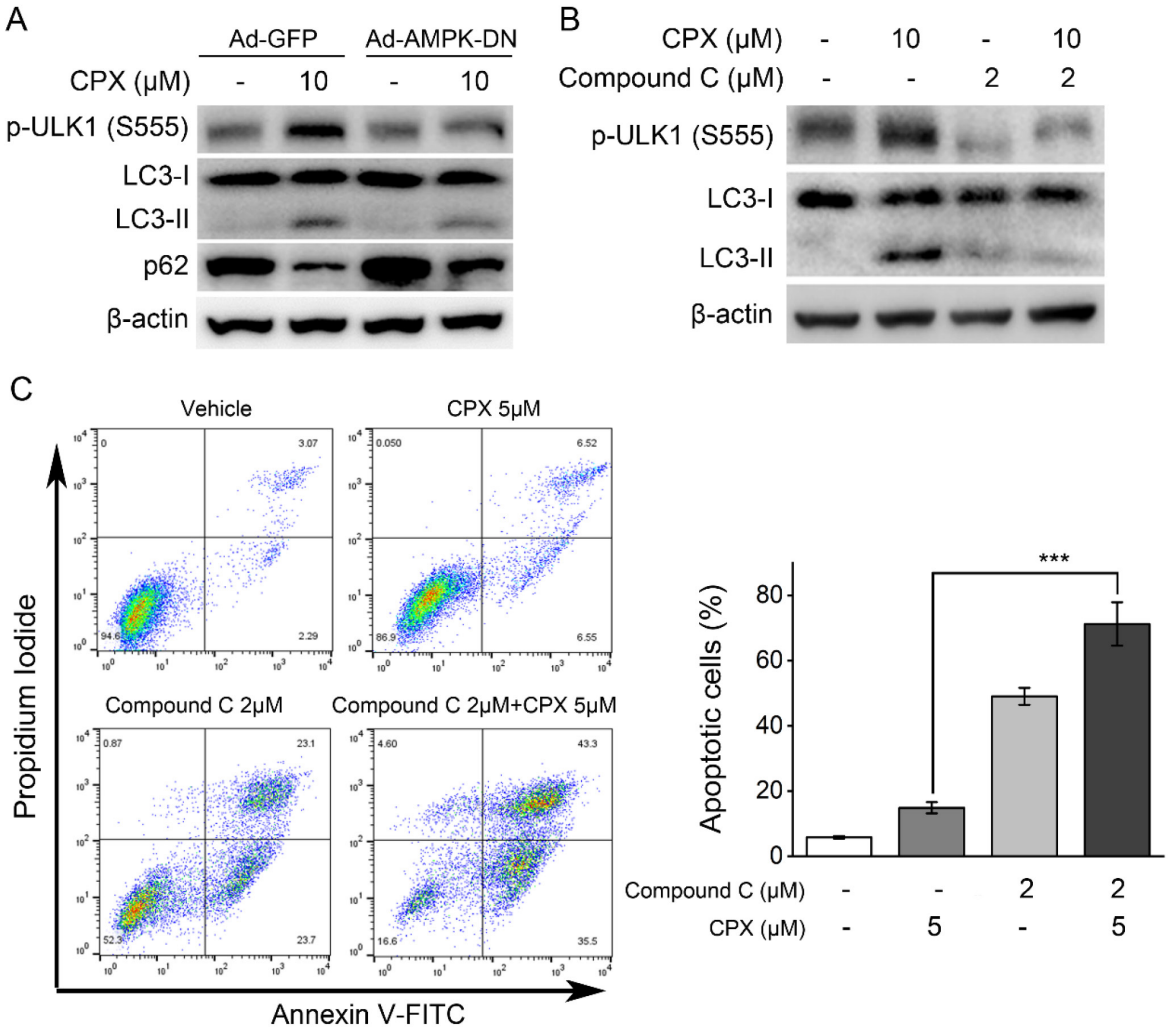
Inhibition of AMPK suppressed CPX-induced autophagy and sensitized K562 cells to CPX. (A) K562 cells were infected with the Ad-AMPK-DN or Ad-GFP (as a control) for 24 h. The cells were then treated with CPX (0, 10 μM) for 24 h, followed by western blot analysis. (B) K562 cells were pretreated with 2 μM of Compound C for 1 h. The cells were then treated with CPX (0, 10 μM) for 24 h, followed by western blot analysis. β-actin was used as a loading control. (C) K562 cells were pretreated with 2 μM of Compound C for 1 h. The cells were then treated with CPX (0, 10 μM) for 48 h, followed by cell apoptosis analysis. Results are presented as mean ± SD (n=3). ***p < 0.001 difference versus 5 μM CPX-treated group.

As demonstrated above, CPX could activate AMPK/ULK1 signaling pathway and induce autophagy in K562 cells. To further investigate the role of AMPK/ULK1 signaling pathway in CPX-induced autophagy, AMPK was inhibited by its inhibitor Compound C or by recombinant adenovirus expressing dominant negative AMPKα (Ad-AMPK-DN), and then the effects of CPX on autophagy in K562 cells were investigated. The results showed that AMPK inhibition by its inhibitor Compound C or Ad-AMPK-DN suppressed CPX-induced ULK1 (Ser 555) phosphorylation, LC3 conversion and p62 decreasing (Figure. 4A and B), suggesting that CPX induced autophagy by activating AMPK-ULK1 signaling pathway in K562 cells. In addition, the effect of AMPK inhibitor Compound C on CPX-induced apoptosis in K562 cells were further investigated. The results showed that Compound C significantly enhanced the effect of CPX on apoptosis induction in K562 cells (Figure. 4C). Taken together, the above results demonstrated that AMPK inhibition rescued CPX sensitivity in K562 cells through autophagy inhibition.

### Autophagy inhibition enhanced the sensitivity of K562 cells to CPX-induced apoptosis

**Figure 5:**
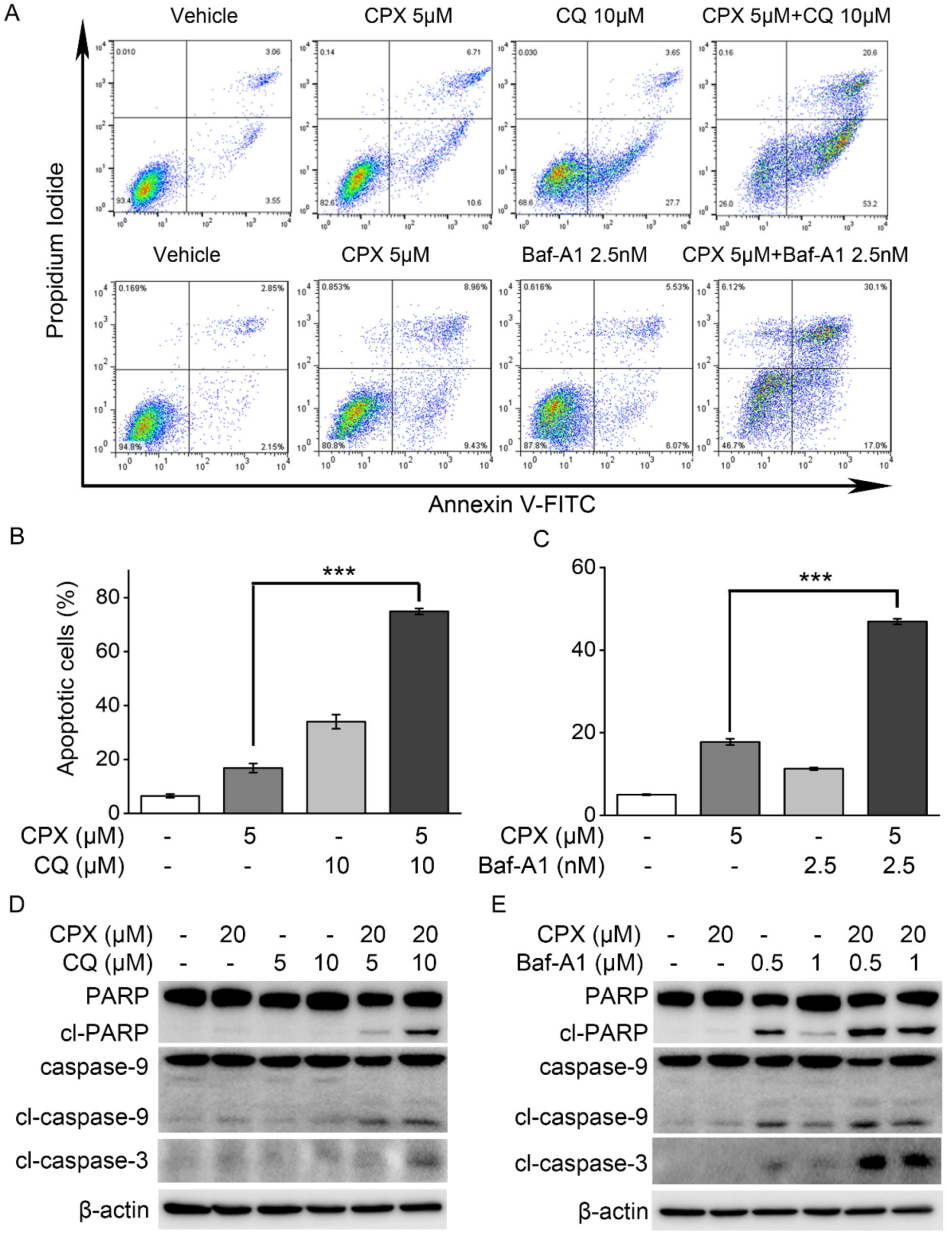
Autophagy inhibition enhanced the sensitivity of K562 cells to CPX-induced apoptosis. (A) K562 cells were pretreated with indicated concentrations of CQ or Baf-A1 for 1 h, and then incubated with or without of CPX for 48 h. The cells were harvested and subjected to apoptosis assay. (B and C) The percent of apoptotic cells was analyzed with FlowJo 7.6.1 software. **p < 0.01, ***p < 0.001 difference versus 5 μM CPX-treated group. (D and E) K562 cells were pretreated with indicated concentrations of CQ (B) or Baf-A1 (C) for 1 h, and then incubated with or without of CPX for 24 h. The cells were harvested and subjected to western blot analysis. β-actin was used as a loading control.

Studies have demonstrated that autophagy may promote cell survival and confer chemoresistance in tumor cells, and that the inhibition of autophagy can effectively potentiate therapy-induced cell death 35, 36. To further confirm the role of autophagy in CPX-induced apoptosis in K562 cells, autophagy was inhibited by using pharmacological inhibitors and determined its impact on cell apoptosis induced by CPX. The results showed the addition of CQ or Baf-A1 significantly increased the level of apoptosis in CPX-treated K562 cells (Figure. 5A-C). Consistently, CPX alone did not induce the cleavage of caspase-3, caspase-9 or PARP in K562 cells, but the combination of CPX with CQ or Baf-A1 resulted in increases in the cleavage of caspase-3, caspase-9 and PARP in K562 cells (Figure. 5D and E), indicating that autophagy activation was responsible for the resistance of K562 cells to CPX-induced apoptotic cell death.

## Discussion

Leukemia is a highly heterogeneous malignant disorder which is characterized by differentiation arrest and unlimited proliferation of abnormal hematopoietic progenitor cells 37, 38. Conventional cytotoxic chemotherapy, such as cytarabine and anthracyclines, has been the main standard of therapy for AML for a long time. In recent years, several targeted small molecules have been developed and approved 37. Some of these agents are currently undergoing preclinical or clinical research 37. The anti-fungal agent CPX, which has been widely used for the treatment of cutaneous fungal infection for over thirty years, was found to have potential anti-tumor activity against a spectrum of human tumors, including leukemia 26, 39, breast cancer 27, 29, pancreatic cancer 40, lung adenocarcinoma 41 and hepatocellular carcinoma 42. In 2009, two independent cell-based chemical screens identified CPX as the top candidate for killing leukemia stem cells 26. Notably, in a phase I clinical study in patients with advanced hematologic malignancies, oral administration of CPX displayed biological activity and no dose limiting toxicity was observed at 40 mg/m^2^ once daily of CPX 24. These preclinical and clinical data support that CPX has a great potential to be repositioned for cancer therapy, especially for hematologic malignancies 24, 25, 42. Mechanistically, similar with the antimicrobial activity, the anticancer effect of CPX is at least partially attributed to the chelation of intracellular iron 26. Interestingly, Eberhard *et al.* reported that in the nonmalignant cell line GMO5757, which was resistant to CPX-induced cell death, CPX chelated intracellular iron as well, suggesting that the sensitivity and the resistance to CPX are not related to its ability to bind intracellular iron 26. Until now, our knowledge of CPX resistance in leukemia and the underlying molecular mechanisms, which is crucial to improve the clinical efficacy of CPX, remains unknown.

In our study, the sensitivity of leukemia cells to CPX was firstly determined in six different human leukemia cell lines by cell viability assay. The result showed that adult acute myeloid leukemia OCI-AML2 cells were the most sensitive to CPX, and human chronic myelogenous leukemia K562 cells were not as sensitive to CPX as the other five leukemia cells. The different response of K562 and OIC-AML2 cells to CPX-induced cell death prompted us to investigate the underlying mechanisms of this difference. Apoptosis, which is also called programmed cell death or type I programmed cell death, can be triggered by two distinct pathways known as the intrinsic (also known as the mitochondrial pathway) and extrinsic pathways 43. Our data showed that CPX effectively induced mitochondrial-mediated apoptosis in OCI-AML2 cells, as evidenced by the decreased ΔΨm and increased cleavage of caspase-9 and PARP, indicating that the inhibitory effect of CPX on OCI-AML2 cells is mainly attributed to the induction of apoptosis. However, CPX induced little apoptosis in K562 cells, suggesting that K562 cells are resistant to CPX-induced apoptosis.

Drug resistance, including intrinsic and acquired resistance, continues to be a major challenge in the fight against leukemia 11. Various studies have reported mechanisms of drug resistance in AML, such as genetic alterations in drug targeting genes, enhanced drug efflux or activated alternative pathways 11. In addition, autophagy can also be activated by chemotherapeutic drugs that might contribute to cell survival and acquired drug resistance in leukemia 44. Autophagy and apoptosis are two distinct mechanisms to deal with the stress of the cells 43. Controllable autophagy is helpful to eliminate tumor cells, and apoptosis induction can prevent the survival of cancer cells and promote cell death. However, dysregulated autophagy will block the activation of apoptosis, allowing tumor cells to survive and promoting chemoresistance. Recent study showed that the activation of autophagy is involved in the resistance of AML cells to the first-line chemotherapy drugs, including Ara-C, daunorubicin and idarubicin 45. Autophagy inhibitor CQ significantly enhanced the sensitivity of these drugs, exhibiting a significant synergy in suppressing cancer cell proliferation and inducing apoptosis 45. Our previous study showed that CPX induced protective autophagy through reactive oxygen species-mediated activation of JNK signaling pathway in human rhabdomyosarcoma cells 46. This promoted us to unravel the role of autophagy in CPX-induced cell death in leukemia. By detecting LC3 conversion (LC3-I to LC3-II) and the protein level of p62, we found that CPX inhibited autophagy in OCI-AML2 cells, but induced high level of autophagy in K562 cells. Thus, we supposed that autophagy activation by CPX may play a protective role in CPX-induced apoptosis in K562 cells.

Since the role of autophagy in cancer and other diseases is prominent and complicated, various autophagy activators or inhibitors have been developed. For example, autophagy inhibitors CQ and Baf-A1 are two useful tools to elucidate the role of autophagy in different disease. CQ, a weak base, can directly inhibit autophagy by accumulating in lysosomes and raise the pH to inhibit lysosomal enzymes 47. Baf-A1 which is a V-ATPase inhibitor can inhibit autophagy by preventing lysosomal acidification 43. In our study, CQ and Baf-A1 were used to clarify the role of CPX-induced autophagy in the resistance of K562 cells to CPX. Our data showed that the combination of CPX with CQ or Baf-A1 synergistically enhanced CPX-induced apoptosis and the cleavage of caspase-3, caspase-9 and PARP in K562 cells, indicating that the resistance of leukemia K562 cells to CPX-induced apoptosis was attributed at least in part to the pro-survival autophagic response. As CQ is a well-known antimalarial drug, our data also suggest that in the future clinical study, the combination of CQ with CPX might be a useful strategy for enhancing the anti-leukemia activity of CPX and achieving improved therapeutic efficacy in chronic myelogenous leukemia.

Moreover, the underlying mechanisms by which CPX induced autophagy were investigated. Autophagy is strictly regulated by various proteins including AMPK, MAPK, phosphoinositide 3-kinase (PI3K)-AKT, Beclin-1 and ATGs 43. Studies showed that ULK1, the most important regulator in autophagy initiation, is regulated via opposing phosphorylation by AMPK and mTOR 19. As a direct substrate of AMPK, ULK1 can be phosphorylated by AMPK at six different sites (S317, S467, S555, T575, S637 and S777) 19. As an important negative regulator, mTORC1 phosphorylates ULK1 on S757 and this phosphorylation disrupts ULK1 and AMPK interaction 19. In the present study, we showed that CPX increased the phosphorylation of ACC, the downstream of AMPK, and induced the phosphorylation of ULK1 at Ser 555, indicating that CPX activated AMPK/ULK1 signaling in K562 cells. The role of AMPK/ULK1 activation in CPX-induced autophagy and CPX resistance was further determined by inhibition of AMPK with a pharmacological inhibitor Compound C and recombinant adenoviruses expressing dominant negative AMPKα, Ad-AMPK-DN. The results showed that the inhibition of AMPK by Ad-AMPK-DN or its inhibitor Compound C decreased CPX-induced autophagy, indicating that CPX induced autophagy by activating AMPK/ULK1 signaling in K562 cells. Moreover, the combination treatment of K562 cells with Compound C and CPX resulted in a significant increase in apoptosis induction in K562 cells. These findings demonstrated that AMPK/ULK1-mediated autophagy played a protective role in CPX-induced apoptosis in K562 cells. Notably, the regulation on AMPK/ULK1 and mTOR/ULK1 signaling by CPX in OCI-AML2 cells was completely opposite to that of K562 cells. How these cellular processes are differentially regulated by CPX between CPX-sensitive and CPX-resistant leukemia cells needed to be investigated in future studies.

## Conclusion

Our present study demonstrates that AMPK-ULK1-mediated autophagy protects human chronic myelogenous leukemia K562 cells from CPX-induced apoptosis. Pharmacological inhibition of autophagy or AMPK re-sensitized K562 cells to CPX-induced apoptosis. These findings provide new insights into the role of AMPK/ULK1-mediated autophagy as a protective mechanism in human chronic myelogenous leukemia K562 cells. Meanwhile, these results suggest that combined treatment of CPX with inhibitors of autophagy or AMPK might be a useful strategy for overcoming CPX resistance, achieving improved therapeutic efficacy in human chronic myelogenous leukemia.
